# Investigation of predictors of increased creatine kinase levels following vascular surgery and the association with peri-operative statin therapy

**Published:** 2009-05

**Authors:** BM Biccard

**Affiliations:** Nelson R Mandela School of Medicine, Durban; Nuffield Department of Anaesthetics, University of Oxford, John Radcliffe Hospital, Oxford, UK; Inkosi Albert Luthuli Central Hospital, Durban, South Africa.

## Abstract

**Summary:**

Although peri-operative statin administration is likely to be cardioprotective, there remains a concern about the risk of rhabdomyolysis and associated renal failure following statin administration in the peri-operative period. The aim of this study was to determine independent predictors of creatine kinase (CK) elevation following vascular surgery.

**Design:**

A retrospective cohort study was conducted. A multivariate analysis using binary logistic regression was conducted of clinical, surgical and laboratory factors which may be associated with a CK exceeding five times the upper limit of normal (ULN).

**Results:**

Four independent predictors associated with a CK > 5 ULN were identified. Statin therapy was protective [odds ratio (OR) 0.096, 95% confidence interval (CI) 0.014–0.68, *p* = 0.019], and a serum creatinine > 180 μmol.l^-1^, positive serum troponins and embolectomy and/or fasciotomy were associated with CK elevation (OR 3.32, 95% CI: 1.03–10.7, *p* = 0.04; OR 5.84, 95% CI: 1.52–22.4, *p* = 0.01; OR 5.62, 95% CI: 1.14–27.8, *p* = 0.03 respectively). Statin therapy was associated with decreased mortality (OR 0.26, 95% CI: 0.08–0.86, *p* = 0.028).

**Conclusion:**

It may be preferable to continue statin therapy in vascular surgical patients even when CK is elevated, as this may decrease mortality if the CK elevation is in the presence of pre-existing renal dysfunction, peri-operative cardiac events or following embolectomy or fasciotomy. Further investigation is required to confirm this observation.

## Summary

Although peri-operative statin administration is likely to be cardioprotective,^1^-^3^ there remains a concern about the risk of rhabdomyolysis and associated renal failure following statin administration in the peri-operative period. Indeed, in the single peri-operative study which examined the association between peri-operative statin use and creatine kinase (CK) levels in vascular surgical patients,^4^ it was shown that over 50% of the patients on statins will have an elevated postoperative CK and 8% will have levels above 10 times the upper limit of normal (> 10 ULN).^4^ Although this incidence is 40 times higher than that reported in the large medical trials,^5^ the incidence of moderate and severe CK elevation did not differ significantly between statin users and non-users in the vascular surgical patients.^4^ On the current peri-operative evidence, we know that the duration of vascular surgery is an independent predictor of CK level,^4^ and that following aortic surgery, CK levels peak at 24 to 48 hours postoperatively.^6^-^7^

However, statin-associated rhabdomyolysis in the peri-operative period is probably rare. It could be estimated at between 0.1% (40 times more frequent than non-surgical patients)^4^,^5^,^8^ and less than 0.5%.^4^ Indeed, even this may be an overestimation of the incidence of peri-operative rhabdomyolyis, as recent meta-analyses of the medical statin trials suggest that it is questionable whether statins actually increase the risk of myalgias,^9^,^10^ CK elevation9 and rhabdomyolysis.^9^-^11^ Hence, unnecessary withdrawal of peri-operative statin therapy (secondary to elevated CK levels) cannot be advocated, as omission of therapy for more than four days postoperatively has also been identified as an independent predictor of cardiac myonecrosis following infrarenal aortic vascular surgery.^12^

There are also limitations associated with basing practice solely on the current literature concerning postoperative CK levels in vascular surgical patients.^4^,^6^,^7^ Firstly, patients with troponin levels above the ULN, or patients with suspected myocardial infarction have been excluded from these analyses.^4^,^7^ Therefore none of these studies evaluated the implications of positive troponin levels in the presence of a high CK^4^,^6^,^7^ on perioperative statin administration. This may have important implications for postoperative management of myocardial infarction. Secondly, the time course of postoperative CK elevation was only derived in aortic surgical patients,^7^ and not from patients undergoing other vascular surgical procedures, and the sample size was only 10 patients.^7^

The aim of this study was therefore to evaluate the pattern and extent of elevation of CK following vascular surgery, to identify possible surgical and medical predictors of CK elevation, and finally to evaluate the role of statin therapy in postoperative CK elevation in patients who underwent elective or urgent vascular surgery.

## Methods

Ethics approval was granted by the ethics committee of the Nelson R Mandela School of Medicine for this study. A retrospective cohort study was conducted using the computerised hospital information system at Inkosi Albert Luthuli Central Hospital. All patients who had vascular surgical procedure between June 2003 and June 2007 were identified. For patients who had had more than one procedure, only the last procedure was analysed. All patients who had had CK levels measured during the hospital admission for surgery were identified. The troponin levels for these patients were also obtained.

The normal values for CK at our laboratory are 32–294 U.l^-1^. A CK < 10 ULN was therefore defined as < 2 940 U.l^-1^. Any patient who had a troponin level above the ULN was classified as ‘troponin positive’. If no troponin levels were above the ULN, the patient was classified as ‘troponin negative’.

## Statistical analyses

All categorical data were analysed using descriptive statistics and either the Fisher’s exact test or Pearson’s Chi-square test, where appropriate. Continuous data were analysed using descriptive statistics and compared using independent samples *t*-test. The postoperative pattern of CK elevation was compared using the Kruskal-Wallis test with Dunn’s multiple-comparisons test, as these data were not normally distributed.

Multivariate analysis was conducted using binary logistic regression analysis. As the sample size for patients with a CK > 10 ULN was only nine subjects, meaningful multivariate analysis of independent predictors for an elevated CK would have been difficult. As a result, a decision was taken to determine independent predictors for a CK > 5 ULN, which would double the sample size. To ensure transparency, the independent predictors identified following multivariate analysis for a CK > 10 ULN, were still determined and included in the article. Clinical risk factors associated with a CK > 5 ULN, which, on univariate analysis had a *p* ≤ 0.25 were entered into the model. A CK > 5 ULN was chosen, as the sample size of patients with a CK > 10 ULN included only nine patients.

A backward stepwise modelling technique was used, based on likelihood ratios with entry and removal probabilities set at 0.05 and 0.1, respectively. The interaction between significant risk factors was also tested. The odds ratio (OR) for a high CK and 95% confidence intervals (CI) are reported. SPSS 15.0 for Windows (6 Sept 2006) was used for all data analysis, with the exception of the Kruskal-Wallis test, which was conducted using GraphPad InStat version 3.06 (2003), GraphPad Software Inc, San Diego, CA, USA.

## Results

Eight hundred and nineteen patients were identified, of whom 114 patients had peri-operative CK levels measured. The medical and surgical risk factors of these patients are shown in Table 1. Patients on statin therapy had a significantly higher incidence of ischaemic heart disease, were more likely to be troponin positive in the peri-operative period, and had significantly less suprainguinal vascular surgery.

**Table 1:** Demographic And Surgical Data Of Patients With Measured Creatine Kinase Levels

*Characteristic*	*On statin therapy (n = 31)*	*No statin therapy (n = 83)*	p*-value*
Pre-operative risk factors				
Age	66 (61–71)	62.5 (55.8–68.3)	0.08*
Ischaemic heart disease	29 (93.5%)	52 (62.7%)	0.001^†^
Serum creatinine > 180 μmol.l^-1^	5 (16.1%)	22 (26.5%)	0.45^†^
Diabetes	14 (45.2%)	27 (32.5%)	0.27^†^
Congestive heart failure	2 (6.5%)	2 (2.4%)	0.30^‡^
Troponin positive	13 (41.9%)	12 (14.5%)	0.004^†^
Peri-operative risk factors				
Supra-inguinal surgery	1 (3.2%)	21 (25.3%)	0.008^‡^
Embolectomy and/or fasciotomy	6 (19.4%)	7 (8.4%)	0.10^‡^
Duration of surgery (minutes)	105 (80–135)	95 (75–158)	0.91*

Values are number (proportion) or median (range). *Mann-Whitney U-test, ^†^Fisher’s exact test, ^‡^Pearson’s Chi-square test.

Of the 114 patients, 45 patients (39%) had a CK level above the ULN. Eighteen patients (16%) had a CK > 5 ULN, of which nine (8%) had a CK > 10 ULN. The CK levels were not normally distributed. Sixteen patients had CK levels measured more than one day pre-operatively or later than four days postoperatively. The median daily and interquartile range for CK levels is shown in Table 2 for the 97 patients who had CK levels measured pre-operatively until the fourth postoperative day. Twenty-five patients (22%) were troponin positive (troponin levels > ULN). Twelve per cent of the troponin-positive patients had a CK > 10 ULN compared to 6.7% for patients who were troponin negative (*p* = 0.389).

**Table 2:** Daily Creatine Kinase Levels For The Vascular Surgical Patients

	*Sample size*	*CK (U.l^-1^) Median (IQR)*	*CK > 5 ULN n (%)*	*CK > 10 ULN n (%)*	*CK > 10 ULN (troponin +) n (%)*	*Sample size (troponin –)*	*CK (U.L^-1^) Median (IQR) (troponin –)*
Pre-operative	10	670 (170–1698)	4 (40)	2 (20)	1 (50)	6	670 (225–2875)
Day 0	29	82 (59–197)	1 (3.6)	1 (3.6)	1 (100)	27	81 (53–181)
Day 1	28	256 (130–505)	6 (21)	5 (18)	3 (60)	20	218 (116–480)
Day 2	7	1455 (119–1519)	3 (43)	1 (14)	0	7	1455 (119–1519)
Day 3	9	525 (327–4043)	3 (33)	3 (33)	1 (33)	7	525 (319–4525)
Day 4	14	444 (185–1378)	7 (50)	3 (21)	0	8	221 (89–2235)

Values are number (proportion) or median (range). IQR interquartile range; ULN upper limit of normal; CK creatine kinase.

The Kruskal-Wallis test showed *p* < 0.0001 between days for both the entire cohort and for the troponin- negative cohort. The Dunn’s multiple comparisons test is shown in Table 3. The third postoperative day showed the largest statistical difference in CK levels when compared to the day of operation, for both the entire cohort and the troponin-negative cohort.

**Table 3:** Dunn’s Multiple Comparisons Test For Perioperative Creatine Kinase Levels

*Days compared*	*Whole cohort p-value*	*Troponinnegative cohort p-value*
Pre-operative vs operative day	< 0.05	NS
Operative day vs 1st postoperative day	NS	NS
Operative day vs 2nd postoperative day	< 0.05	< 0.05
Operative day vs 3rd postoperative day	< 0.001	< 0.01
Operative day vs 4th postoperative day	< 0.05	NS

NS not significant (*p* > 0.05)

Univariate analysis of factors associated with a CK > 5 ULN are shown in Table 4. Only a serum creatinine > 180 μmol.l^-1^ was significantly associated with a CK > 5 ULN.

**Table 4:** Univariate Analysis Of Medical And Surgical Risk Factors Associated With A Creatine Kinase > 5 ULN

*Risk factor*	*CK < 5 ULN (96 patients)*	*CK > 5 ULN (18 patients)*	p*-value*
Ischaemic heart disease	69 (72%)	12 (67%)	0.778*
Diabetes	37 (39%)	4 (22%)	0.284*
Serum creatinine > 180 μmol.l^-1^	19 (20%)	8 (44%)	0.030^†^
Troponin positive	18 (19%)	7 (39%)	0.058^†^
Statin therapy	29 (30%)	2 (11%)	0.095^†^
Supra-inguinal surgery	19 (20%)	3 (17%)	0.758
Embolectomy and/or fasciotomy	9 (9%)	4 (22%)	0.116^†^
Duration of surgery	100 (68–145)	85 (64–138)	0.306^‡^

Values are number (proportion) or median (range). CK creatine kinase; ULN upper limit of normal *Fisher’s exact test; ^†^Pearson’s Chi-square test; ^‡^Mann-Whitney U-test

The multivariate analysis of independent predictors of elevated CK levels are shown in Table 5. Embolectomy and/or fasciotomy were an independent surgical factor and an elevated serum creatinine was an independent medical risk factor. Statin therapy was associated with significantly less CK elevation for the entire cohort. There was no interaction between the independent variables.

**Table 5:** Independent Predictors Of An Elevated Creatine Kinase On Multivariate Analysis

*Creatine kinase level*	*Variable*	*OR (95% CI)*	p*-value*
CK > 5 ULN (whole cohort)	Statin therapy	0.096 (0.014–0.68)	0.019
	Creatinine > 180 μmol.l^-1^	3.32 (1.03–10.7)	0.044
	Troponin positive	5.84 (1.52–22.4)	0.010
	Embolectomy and/or fasciotomy	5.62 (1.14–27.8)	0.034
CK > 5 ULN (troponin negative)	Embolectomy and/or fasciotomy	10.3 (1.49–71.5)	0.018
CK > 10 ULN (whole cohort)	Embolectomy and/or fasciotomy	8.6 (1.38–54.2)	0.021
	Creatinine > 180 μmol.l^-1^	5.37 (1.14–25.4)	0.034
CK > 10 ULN (troponin negative)	Creatinine > 180 μmol.l^-1^	13.0 (1.21–140)	0.034

OR, odds ratio; CI, confidence interval; CK, creatine kinase; ULN, upper limit of normal.

All-cause mortality was reported in 33 (29%) of the 114 patients. An elevated CK was not found to be a univariate predictor of mortality (CK > 5 ULN, OR 1.94, 95% CI: 0.76–4.94, *p* = 0.17). Multivariate analysis of the entire cohort was conducted, entering medical, surgical and CK variables into the model. Statin therapy was the only independent predictor of mortality. Statin therapy was protective with an OR 0.26, 95% CI: 0.08–0.86, *p* = 0.028 for all-cause mortality.

## Discussion

This study presents a number of important findings that may influence peri-operative clinical practice. Firstly, although statin therapy may be associated with an elevated CK in vascular surgical patients, there are a number of other factors associated with an elevated CK, including the surgical procedure (embolectomy, fasciotomy and aortic surgery^7^), the duration of surgery,^4^ the medical condition of the patient (renal dysfunction) and complicating cardiac events. These factors may be potentially more important for patient outcome than the rare occurrence of statin-associated rhabdomyolysis. Indeed this study suggests that the administration of statin therapy in these patients, even with an elevated CK, is preferable and is independently associated with decreased peri-operative mortality. It is important to appreciate that this is an association however, and only an appropriate randomised, controlled trial would be able to determine if this has a causal effect.

This study suggests that withdrawal of statin therapy in these vascular surgical patients based on an elevated CK level alone may adversely affect the patient’s outcome, both because statins may be protective (as shown in this study) and withdrawal is associated with myonecrosis.^12^ Withdrawal of statin therapy in vascular surgical patients with an elevated CK, in the presence of an elevated serum creatinine, following embolectomy and/or fasciotomy or in the presence of elevated troponins may therefore be inappropriate. It is important to realise that this study unfortunately could not confirm whether this association is also present in patients with a CK > 10 ULN.

The second important finding is that embolectomy and/or fasciotomy was independently associated with an elevated postoperative CK level. Therefore, following aortic surgery,^7^ embolectomy or fasciotomy, these surgical factors should be considered as the primary cause of any postoperative elevation in the CK level. Importantly, CK levels exceeding 50 ULN have been identified in other situations associated with muscle damage, such as excessive exercise, often with no serious complications.^13^

What may be the possible mechanisms related to an improved outcome associated with statin therapy in vascular patients requiring embolectomy or fasciotomy? Statins may theoretically be protective for the following reasons. Firstly, approximately two-thirds of patients presenting for embolectomy for acute arterial occlusion have associated atrial fibrillation.^14^ It is possible that statin therapy may improve cardiac outcome if this is associated with an acute coronary event.^15^ Secondly, as statins improve plaque stability,^16^ it is possible that the volume of vulnerable plaque is less in patients on statin therapy, and this may decrease the morbidity associated with embolectomy and fasciotomy.

The third important finding is the time to peak CK elevation. This study suggests that there may be a difference in time to peak CK levels following supra- versus infra-inguinal vascular surgery. Although Andersen *et al*. showed that following aortic surgery, CK levels peaked at 48 hours,^7^ this study suggests that in patients undergoing predominantly infra-inguinal vascular surgery, CK peaks 72 hours postoperatively. This finding is important, as it has been suggested that if CK is > 10 ULN postoperatively, provided that a fall in CK levels is documented by 72 hours, it is probably reasonable to continue statin therapy.^17^ However, this study suggests that in patients undergoing predominantly infra-inguinal vascular surgery, the postoperative CK peak may be slightly later, at 72 hours. Therefore, the clinical finding of a CK > 10 ULN at 72 hours may reflect the natural peak following infra-inguinal surgery, and it may be prudent then to continue statin therapy until 96 hours postoperatively, provided a decline in CK levels is then documented and there are no associated muscle symptoms.^13^

The fourth finding of importance was the association between an elevated serum creatinine and a raised CK level. Although randomised medical trials have shown no evidence that serum creatinine is associated with an increased CK level,^18^ it is important to note that a raised serum creatinine is consistently an important independent predictor of peri-operative cardiac events,^19^-^21^ and indeed it is these patients who are most likely to benefit from peri-operative statin therapy. Therefore, although no interaction was shown between patients who were troponin positive and those with an elevated serum creatinine in this study, it is possible that in a study with a larger sample size, interaction may be shown between these two variables. This indeed would explain why statin therapy was protective in this study, even in the presence of an elevated CK. It is therefore suggested that a raised CK in a vascular surgical patient with renal dysfunction should probably be considered an indication to continue the statin therapy, as opposed to withdrawing it.

Finally, contrary to expectations, CK levels were less likely to exceed 5 ULN in patients on statin therapy in this study, even though the statin group included significantly more troponin-positive patients.

## Limitations

This study was a retrospective observational study, and hence there was bias in the selection of which patients had peri-operative CK and troponin levels measured. It is likely that a number of these patients had these investigations on the clinical suspicion of a major cardiac event. However, conversely, this could be considered a strength of this study, as it is these patients in whom statins may be advocated and indeed in whom statins are potentially protective.^1^-^3^ It is these patients in whom withdrawal of statin therapy may be associated with an adverse outcome. Importantly, in this study, statin therapy in these patients was associated with improved survival and lower CK levels.

Despite the possible protection of statin therapy against a CK > 5 ULN, shown in this study, unfortunately due to the small sample size of patients with a CK > 10 ULN, it was impossible to confirm firstly, whether statin therapy is independently associated with fewer patients with a CK > 10 ULN, secondly, whether troponin elevation is an independent predictor of a CK > 10 ULN, and finally whether continuation of statin therapy is protective in vascular surgical patients whose CK exceeds 10 ULN. This is certainly an issue that needs further investigation, as unnecessary withdrawal of statin therapy in vascular surgical patients will increase cardiac morbidity.^12^

## Conclusion

Statin therapy was associated with lower CK levels in vascular surgical patients who had renal dysfunction, peri-operative cardiac events and following embolectomy and/or fasciotomy. Continuation of statin therapy despite elevated CK levels in these patients may have resulted in an improved survival. Further investigation is required to confirm this observation and determine whether it is also true of patients who have a CK > 10 ULN.
